# P24 Ankylosing spondylitis & uveitis - challenges in management

**DOI:** 10.1093/rap/rkab068.023

**Published:** 2021-10-19

**Authors:** Katarzyna Nowak, Michelle McHenry, Clara McAvoy

**Affiliations:** 1 Musgrave Park Hospital, Belfast, United Kingdom; 2 Royal Victoria Hospital, Belfast, United Kingdom

## Abstract

**Case report - Introduction:**

Ankylosing spondylitis is a chronic inflammatory condition involving spine, chest and sacroiliac joints. It can be associated with extra spinal manifestations which include uveitis and enthesitis, as well as other features of an inflammatory spondyloarthropathy including psoriasis and inflammatory bowel disease. It is characterised by the presence of inflammatory back pain, limited range of spinal movement, sacroiliitis on imaging, excess spinal bone formation and a high prevalence of HLA-B27. The main aims of treatment are to improve symptoms and retain function as well as to prevent disease complications.

**Case report - Case description:**

A 49-year-old male diagnosed with ankylosing spondylitis at the age of 23 (HLA-B27 positive, classical radiographic features on plain radiographs and MRI), with background of meningitis and subsequent epilepsy (not on treatment since the age of 13), severe hip osteoarthritis and osteoporosis. He has peripheral elbow and knee synovitis. At the age of 27 he developed sight-threatening bilateral recurrent anterior uveitis complicated by cystoid macular oedema (causing central vision loss). Initial management included anti-inflammatories and steroids. He progressed to biologics but developed a rash on adalimumab. He was switched to infliximab in 2010. In 2015 it was switched due to reduced response to golimumab which was non-beneficial. Subsequently he was switched to etanercept but after 3 months it was discontinued due to eye flare. The patient’s therapy was switched to certolizumab, which was non-beneficial for spine and discontinued. In 2016, infliximab was retried as the patient thought it worked best. Unfortunately, in 2018 patient developed Infliximab antibodies and treatment was changed to secukinumab. After 9 months this was stopped owing to ongoing active eye and spine disease. Adalimumab was re-tried; however, it was discontinued as it was showing no benefit. In 2020 Eeanercept (Benepali) was re-tried; however, only for 4 months because of severe flare of joint and eye disease. Tofacitinib was commenced but was non-beneficial and hence it was discontinued. Currently he continues on ixekizumab, oral prednisolone and also receives steroid eye and joint injections. On commencing biologics he was on methotrexate; however, the patient stopped. He was switched to mycophenolate mofetil which he remained on for 10 years. It was switched to methotrexate to try and control peripheral inflammatory joint disease better (re-tried for the second time) which the patient has recently discontinued as he felt it was non-beneficial.

**Case report - Discussion:**

The case is very challenging. The patient’s disease continues to flare despite changing therapies. He continues to get flares in his back disease, eye disease and peripheral synovitis. His latest BASDI was 5.1 and VAS was 9/10. Measurements: intermalleolar distance 15cm, modified Schober’s 4cm, right lateral flexion 3.8cm and left lateral flexion 11cm. His latest MRI of whole spine and SI joints (in 2020) showed signs of previously active seronegative spondyloarthropathy with established cervical and thoracic ankyloses as well as bilateral sacroiliac joint ankylosis. There is a need for repeated steroid injections to the knees as well as the eyes. He continues on oral prednisolone which was first started in 2006. This is associated with multiple side effects, i.e., osteoporosis. The patient has already completed 8 years of bisphosphonate therapy. He is under regular review with the osteoporosis clinic. His bone density scan showed worsening bone mineral density and bisphosphonates had to be restarted. The patient adjusted medications depending on his symptoms. Recently he also discontinued methotrexate as he felt it was of no benefit to him at all. We are very limited at present to find combined medications to manage both the patient’s inflammatory back disease and inflammatory eye disease.

**Case report - Key learning points:**

This patient was diagnosed with ankylosing spondylitis 26 years ago. To date he has tried 5 different anti-TNF agents (two of which have also been re-tried at a different time point). This failed to control his disease as he continued to experience flares in either inflammatory back or eye disease. The biologic classes were also changed – he has tried one IL-17A agent as well as a JAK-I. Unfortunately, they both failed to control his disease. He currently continues on a second IL-17A; however, he still requires steroid eye and joint injections as well as systemic steroids. Throughout the years he has tried different DMARDs – most recently methotrexate. Unfortunately, the patient decided to discontinue it as he felt it was of no benefit to him. All of the above indicates that there is still a need to find a collaborative treatment option that will control the patient’s inflammatory back disease, inflammatory eye disease and peripheral synovitis. The aim would be, if possible, to decrease the steroid dose as well (currently on prednisolone 15mg daily) in order to prevent the steroid-associated side effects.

